# The Effect of Three-Year Swim Training on Cardio-Respiratory Fitness and Selected Somatic Features of Prepubertal Boys

**DOI:** 10.3390/ijerph19127125

**Published:** 2022-06-10

**Authors:** Ryszard Zarzeczny, Mariusz Kuberski, Edyta Suliga

**Affiliations:** 1 Institute of Health Sciences, Collegium Medicum, Jan Kochanowski University, 5 Żeromskiego Str., 25-369 Kielce, Poland; edyta.suliga@ujk.edu.pl; 2 Chair of Physical Culture Sciences, Jan Długosz University in Częstochowa, 13/15 Armii Krajowej Str., 42-200 Częstochowa, Poland; m.kuberski@ujd.edu.pl

**Keywords:** cardio-respiratory fitness, 20 m shuttle run test, physical traits, physical activity, swim training, prepubertal boys

## Abstract

The data regarding somatic and physiological effects of sport-related physical activities in youth are limited. Moreover, whether exercise training is capable of increasing cardio-respiratory fitness remains a disputable issue. The study undertook to assess the effect of swimming training on cardio-respiratory fitness (CRF) and the development of physical traits in prepubertal boys, and to determine which of the traits is the best predictor of their CRF. Forty 10-year old prepubertal boys (10.5 ± 0.3 y) were divided into two groups (swimmers (SG), *n* = 20, and controls (CG), *n* = 20), which underwent anthropometric measurements and performed a 20 m shuttle run test (20 mSRT) semi-annually over a 3-year period. CRF indices (the number of 20 mSRT shuttles, maximal speed, and VO_2_max) were higher overall in the SG compared with the CG (*p* < 0.001). The values of the main physique variables increased faster in the CG, but the groups showed no differentiation of physical traits. In both groups, CRF indices were associated with the participants’ physical traits, the most strongly with the sum of four skinfold thicknesses in the SG and knee breadth in the CG. These results suggest that swimming training is a form of additional physical activity that improves prepubertal boys’ CRF but does not significantly affect their physical development. In using the 20 mSRT to assess the CRF of prepubertal boys, their physical activity level and age-related changes in body fatness need to be considered.

## 1. Introduction

Physical activity has long been known to significantly benefit human health and wellbeing [1]. In adults, high physical activity levels considerably reduce the risk of cardiovascular diseases, osteoporosis, and colon cancer, prevent or delay the development of high blood pressure, help manage body mass and blood glucose concentration, improve cardio-respiratory fitness (CRF), and alleviate depression and anxiety symptoms [2]. Similar benefits of regular physical activity are also observed in children and adolescents [3], although its association with their future health appears less distinct than in the case of adults [4]. There is, however, sufficient evidence of exercise being able to reduce the risk of obesity and related conditions to promote it among children and adolescents [5].

The importance of physical activity for children and adolescents has been highlighted in two recent publications: a report by Guthold et al. [6] and the WHO Guidelines on Physical Activity and Sedentary Behaviour [7]. After carrying out a pooled analysis of 298 population-based surveys with 1.6 million participants, Guthold et al. [6] concluded that 81% of the global adolescent population (ages 11–17 years) were insufficiently physically active. The finding prompted them to call for an urgent scaling up of policies and programs addressing adolescents’ health needs. Additionally, in 2020, WHO published the Guidelines on Physical Activity and Sedentary Behaviour [7], recommending that 11–17-year-olds should reduce sedentary time, engage in an average of 60 min/day of moderate-to-vigorous intensity aerobic physical activity each week, and to perform regular muscle-strengthening exercises. The guidelines also stressed that future research should address problems such as the lack of knowledge about how physical activity and the development of motor skills are related to each other, or the insufficient evidence available to determine whether the relationship between physical activity and its health outcomes for children and adolescents depends on the type or domain of physical activity.

A physical activity that is very popular with children and adolescents is swimming, probably because of its various benefits, including physiological (enhancement of overall physical fitness), psychological (greater self-confidence, lower anxiety and stress), and social (peer-group interaction, inclusion, safety). Swimming also improves the motor functions (speed, agility, perceptual-motor function), cardio-respiratory fitness, muscular strength, endurance, and flexibility. It can also help with weight management, and the risk of injury is relatively low compared with other sports [8]. All these benefits make swimming a recommendable physical activity for people of all ages and health conditions.

Because swimming is significantly different from other sports (the horizontal position of the swimmer in water causes different gravitational and resistive forces to operate and a different pattern of respiration is necessary), its health outcomes for children and adolescents, such as CRF or physical development, can also be different from those offered by other types of exercise.

Following the long-term athlete development (LTAD) model, a widely used training strategy stressing the need to develop athletes’ fundamental motor abilities at the optimal physical development stage, the focus of swimming training for children and adolescents is on aerobic capacity [9]. This leads to an interesting question about whether, and in what respects, the CRF of children and adolescents who practice swimming differs from that of their untrained peers. Answering the question could resolve at least some of the controversies about the ability of physical training to improve aerobic capacity in adolescents [10].

Studies show that the top adult swimmers are usually taller and have longer upper and lower limbs and greater biiliocristal and wrist breadths than swimmers with inferior time performance [11,12,13]. Similar differences are also observed among young swimmers.

The solid evidence of positive correlations between swimming performance and the swimmers’ body height, arm span, upper and lower limb length, and wider girths [14] contrasts with the limited knowledge of associations between the physical traits and CRF of young swimmers. It is also noteworthy that the knowledge comes from cross-sectional studies on young swimmers who had been recruited by clubs based on their physical and physiological characteristics; as a result, the effects of regular swimming training and natural biological development are hard to distinguish between.

Considering the findings of the cited studies and the knowledge that physical activity tends to decline throughout adolescence [15], this study was undertaken (1) to assess the CRF and the development of physical traits of prepubertal boys participating in a 3-year swimming training program and to compare them with those of their untrained peers, and (2) to determine which physical trait would be the best predictor of the swimmers’ and controls’ CRF.

## 2. Materials and Methods

### 2.1. Participants

The study participants were boys who had swimming lessons as part of the physical education (PE) program (45 min, twice a week) in grades 1–3 of elementary schools in Częstochowa (Poland). They were recruited to the study on a voluntary basis at age 10 (mean ± SD: 10.5 ± 0.3 years), after they finished the third grade and were invited to join the swimming clubs at their schools without any preselection procedure. Twenty of those who had accepted the invitation were allocated to the swimming group (SG) in the study. The control group (CG) was formed of twenty boys of the same age who declared that they did not participate in any physical activity beyond PE classes. Both groups continued to participate in regular PE education classes (on land) in the higher grades.

The biological age of the participants was determined by calculating their maturity offset (MO) based on their age and body height, according to the formula proposed by Moore et al. [16].

The boys and their parents were informed of the purpose and methodology of the study and gave written consent to participate in it, as required by the Declaration of Helsinki. The protocol of the study was approved by the Bioethics Commission.

### 2.2. Design and Procedures

After enrolment, and then every six months (in April and October) over the next three years, the boys’ resting heart rate (HR), anthropometric features, skinfold thicknesses, and CRF indices were measured between 08:00 a.m. and 12:00 p.m.

Resting heart rate (HR_rest_) was measured in the lab after 15 min of rest in a sitting position (to avoid confounders such as previous exertion, brisk walk or anxiety) by palpating the carotid artery and taking a pulse count for 15 s and multiplying the result by 4.

Anthropometric measurements, which were also taken in the laboratory, included body mass and height, the length of the upper (acromiale-dactylion) and lower (femur and tibia, i.e., trochanterion-sphyrion tibiale) limbs; shoulder breadth (biacromial breadth), elbow breadth (bicondylar, humerus breadth), hip (biiliocristal breadth), knee (bicondylar, femur breadth); the girths of the head (perpendicular to the long axis of the head), chest (at the level of the mesosternale landmark and perpendicular to the long axis of the thorax), relaxed arm (at the level of the mid-acromiale-radiale), abdomen (waist) (at the narrowest point between the lower costal border and the iliac crest), and thigh (1 cm below the gluteal fold, perpendicular to the long axis of the thigh). Body mass and height measurements were performed using a standard electronic scale with a stadiometer (WPT 150.0; RadWag, Radom, Poland) with an accuracy of 0.1 kg and 0.5 cm, respectively. Participants’ BMIs (Body Mass Indexes) were calculated by dividing their body mass by the square of the body height (in meters). Other measurements were taken with an anthropometry tape and small and large sliding calipers by an experienced anthropometrist on the right side of the participant’s body in the Frankfort plane, as recommended by the International Society for the Advancement of Kinanthropometry (ISAK) [17].

Skinfold thickness measurements involved the subscapular skinfold (at the inferior angle of the right scapula), biceps skinfold (at the upper arm mid-point mark on the anterior surface of the right upper arm), triceps skinfold (at the upper arm mid-point mark on the posterior surface of the right upper arm), and suprailiac skinfold (at the iliac crest skinfold site). The measurements were taken by an experienced anthropometrist on the right side of the participant’s body in the Frankfort plane with Harpenden calipers (M2 TOP, Käfer Messuhrenfabrik GmbH & Co. KG, Villingen-Schwenningen, Germany) as per the ISAK guidelines [17]. Subscapular and triceps skinfolds were used to calculate the percentage of body fat for each participant, according to the formula given by Slaughter et al. [18]:

When the sum of the triceps and subscapular is less than 35 mm:

body fat (%) = 1.21 × (triceps + subscapular) − 0.008 × (triceps + subscapular)^2^ × 1.7

When the sum of the triceps and subscapular exceeds 35 mm:

body fat (%) = 0.783 × (triceps + subscapular) + 1.6

Participants’ CRF was assessed by means of a 20 m shuttle run test (20 mSRT) [19], which was performed in the school gymnasium at a temperature between 19 and 21 °C. The test required the participants to run back and forth between two lines 20 m apart at a speed dictated by pre-recorded audio beeps, which were checked for accuracy prior to testing. The initial speed of 8.5 km × h^−1^ was increased with consecutive 1 min stages by 0.5 km × h^−1^. Each stage consisted of multiple “shuttles”, whose number increased with speed. The participants were grouped to provide a competitive environment and were instructed to keep running at the pace of the beeps for as long as possible. The test was terminated when a subject could no longer keep pace with the beeps (failed to complete two consecutive shuttles in time) or when fatigue prevented him from continuing the test. Because running speed is initially very low, no warm-up was administered. Participants’ CRF was evaluated based on the number of shuttles they completed during the 20 mSRT, maximum 20 mSRT speed (the speed for the last completed stage), and maximum oxygen uptake (VO_2_max) estimated taking into account the participant’s calendar age and maximum 20 mSRT speed. The following formula was used to this end [19]:

VO_2_max = 31.025 + 3.238 × S − 3.248 × A + 0.1536 × S × A

where S is maximum 20 mSRT speed; A is participants’ calendar age.

### 2.3. Physical Education (PE) Classes

The PE classes were conducted according to the governmental core curriculum for physical education in grades IV–VIII of a primary school. This core curriculum is comprised of teaching the basic elements of different kinds of sports (team games, track and field, gymnastics, etc.) and their improvement in the higher grades. Generally, the PE classes consisted of an introduction to the lesson and warm-up (10–15 min), a main activity block (20–25 min), and a warm-down (5–7 min). The ratio between low intensity exercises and moderate-to-vigorous intensity exercises was 60% to 40%.

### 2.4. Additional Physical Activity

In addition to regular PE classes (on land), boys in the swimming group trained four times in a week over the three years of the study in their schools’ swimming pools in the morning hours. Training sessions of 70 min consisted of a warm-up with stretching exercises on land and a 200–400 m front crawl and back-stroke swimming, a main training block during which the boys swam several 400 m swims to practice technical swimming skills and improve their aerobic capacity, and a warm-down involving stretching exercises on land. The ratio between aerobic exercises and anaerobic exercises during a session was 80% to 20%. The distances the participants swam during a training session in each of the three years of observation were approximately 1500 m, 2000 m, and 2500 m, respectively.

### 2.5. Statistical Analysis

Data were tested for normality of distribution using a Shapiro–Wilk test. When distributions were not normal, they were transformed into logarithms for further analysis. The statistical significance of differences between swimmers’ and controls’ variables was determined using a two-way, repeated measures ANOVA with one factor (time). To better present the patterns of changes in the variables (interaction effect), the slope of linear regression was calculated based on the variables’ means obtained during consecutive semi-annual measurements. The effect of swimming training on swimming time was determined by performing one-way ANOVA with repeated measures (time) on the results of the 400 m front crawl test. The outcomes of one- and two-way ANOVA were subjected to a post-hoc analysis with the Neuman–Keuls test. The relationships between CRF indices (the number of shuttles achieved by a participant during the 20 mSRT, maximum 20 mSRT speed, and VO_2_max) and selected somatic variables were assessed by Pearson’s product moment correlation coefficients. To avoid the occurrence of type-1 error associated with multiple comparisons, the Benjamini–Hochberg procedure and a False Discovery Rate of 0.1 were used as proposed by McDonald [20]. Each variable’s effect on participants’ CRF indices was assessed by means of a stepwise multiple regression analysis with backward elimination. Only variables that significantly correlated with the dependent variable were included in the analysis. All computations were performed in Statistica 12.0 (Statsoft, Krakow, Poland).

The results are presented as arithmetic means and standard deviations (±SD) or as medians (M) and interquartile ranges (IQR) when their distributions were not normal. The level of statistical significance is *p* < 0.05 for all cases excluding multiple comparisons (the Benjamini–Hochberg procedure).

## 3. Results

According to maturity offset, all boys were at the prepubertal phase of development during the study (Table 1). There was no statistically significant difference between the swimmers and the controls at the baseline in any variable (Table 1).

The end-point measurements showed that the values of most variables, excluding % body fat, biceps skinfold thickness, triceps skinfold thickness, and resting HR, increased significantly from the baseline (a significant main effect of time). The post-hoc analysis revealed many instances of significant increases between consecutive measurements (Table 1).

A significant main effect of between-group differences was only determined for CRF indices (the number of shuttles during the 20 mSRT, maximum 20 mSRT speed, and VO_2_max determined from the 20 mSRT results). However, the post-hoc analysis of consecutive measurements did not show the swimmers’ and controls’ CRF to be significantly different for any of them (Table 1).

The effect of interaction proved to be statistically significant for 15 out of 26 variables analyzed. Generally, the linear regression slope values of the somatic variables were greater in the CG than in the SG. The same pattern was found for body fatness indices, except that in the CG they did not change much from the baseline (mainly increased), whereas in the SG most of them were negative. Additionally, the values of CRF indices in the CG increased more slowly than in the SG. The slope of resting HR did not change in the CG over the study period; in the swimmers, it was consistently negative (Table 1).

**Figure 1:**
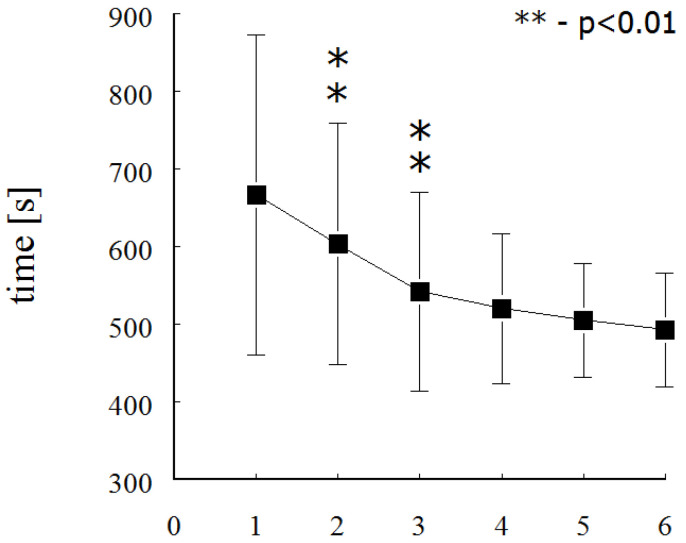
The results of 400 m front crawl swim test in 6 consecutive measurements in swimming group. The asterisks denote a statistical difference from the previous measurement.

The effectiveness of additional physical activity is shown in Figure 1. Over the 3 years of the study, boys in the SG improved their 400 m front crawl swim time progressively and statistically significantly (F = 20.182; *p* < 0.001).

In Table 2, Table 3 and Table 4, Pearson’s correlations between the somatic variables and CRF indices corrected for multiple comparisons using the Benjamini–Hochberg procedure are presented. Generally, the SG had more somatic variables that significantly correlated with CRF indices than the CG. None of the body fatness variables in the latter group was found to be significantly associated with any of CRF indices. In both study groups, the number of shuttles achieved by participants during the 20 mSRT and maximum 20 mSRT speed were significantly correlated with their chronological age, elbow breadth, biiliocristal breadth, and biacromial breadth (Table 2 and Table 3). However, the groups differed entirely regarding variables correlated with VO_2_max (Table 4).

Because many correlations between participants’ somatic features and CRF indices were statistically significant, and because the correlations differentiated the controls from the swimmers, a stepwise multiple regression analysis was applied to see which of the somatic variables would best predict the values of CRF indices. In the CG, the only independent variable to be statistically significantly associated with CRF indices (dependent variables) was knee breadth, which explained 22.8% of the variability in the number of shuttles achieved during the 20 mSRT, 23.0% of the variability in the maximum 20 mSRT speed, and 6.7% of the variability in VO_2_max (Table 5). Interestingly, in the CG, of all somatic features only knee breadth was statistically significantly associated with the participants’ VO_2_max (Table 4). As for the swimmers, statistically significant correlations between somatic features and CRF indices were determined for the sum of four skinfold thicknesses (subscapular, biceps, triceps, and suprailiac) (Table 5). The sum of four skinfold thicknesses explained 17.6% of the variability in the number of shuttles achieved during the 20 mSRT, 16.6% of the variability in the maximum speed at the last completed 20 mSRT stage, and 18.5% of the variability in VO_2_max (Table 5).

Because knee breadth and the sum of four skinfold thicknesses best predicted the values of CRF indices in controls and swimmers, a multiple regression analysis was carried out to determine which of the CRF indices (treated as independent variables) would be the best predictor of knee breadth and the sum of four skinfold thicknesses (treated as dependent variables) in the controls and the swimmers, respectively (Table 6). It pointed out that these were the maximum 20 mSRT speed in the CG and VO_2_max in the SG (Table 6).

## 4. Discussion

The objectives of this study were to determine how the CRF and somatic growth of prepubertal boys engaged in additional physical activity, specifically three years of swimming training, would change compared with same-age untrained controls, and which somatic trait would be the best predictor of the CRF in both groups. The study demonstrated, that while none of the somatic variables measured semiannually significantly differentiated the swimmers from the controls, the values of some of them increased faster in the control group. Even so, the three-year values of CRF indices were higher for the swimmers than for the controls (a significant between-group effect). The best predictors of participants’ CRF indices proved to be sum of four investigated skinfold thicknesses (the swimmers) and knee breadth (the controls).

Studies comparing young swimmers and controls show that the former are taller and heavier and have broader shoulders [21,22]. Similar differences are found between boys with different swimming skills. In a ranking of young swimmers based on their best 100 m freestyle swim times, the leaders were taller, heavier, and had longer limbs than the other athletes [23]. The absence of such differences between the swimmers and the controls reported by some authors [24] is consistent with none of the consecutive measurements in our study finding differences between the anthropometric traits of the swimmers and the controls. There are two likely reasons for this: (1) the young swimmers we studied were not required to meet any special anthropometric and physiological criteria to be accepted by their sports clubs (as is the usual case); (2) our study was a longitudinal experiment whereas other studies used a cross-sectional approach.

The interaction effect revealed by two-way ANOVA in this study is interesting in that it shows that the values of 7 out of the 14 somatic variables examined (upper and lower limb lengths, elbow breadth, knee breadth, biacriomial breadth, biiliocristal breadth, and thigh girth) increased more in the controls than in the swimmers over the three years of the study period. An explanation of this can be found in many works on the effect of exercise training intensity on human biological development, according to which it can be slowed down by high exercise loads [25]. Research has shown that the maturity of the swimmers expressed by their somatic features is above normal, which is explained by the criteria they need to meet to enter this sport [26]. The results of our study suggest that although maturity offset did not significantly differentiate the swimmers from the controls, the rate of biological development (determined from changes in above mentioned somatic variables) was lower in the former. There are two possible explanations for why the interaction effect was not significant for body height while being significant for the lower limb length. One is that the trunk develops more slowly compared with the sub-ischial length [27], and the other is the special nature of swimming training (as the spine is less loaded during a horizontal position in water, in the swimmers it can be slightly longer than in the controls).

An increase in the CRF of prepubertal children, especially VO_2_max, is a controversial matter [28]. According to the study of Krahenbuhl et al. [29], the relative VO_2_max of prepubertal boys who do not engage in any additional physical activity is rather constant until they reach puberty [29]. In those who do endurance training, it may be higher by approximately 5–6%, or even by 8–10%, according to studies where the effect of endurance training proved to be significant [10]. These values are broadly comparable with 13.2% for the swimmers in our study (in the controls, VO_2_max of increased by 1.8% over the three-year period). The physiological evidence of the swimmers’ adaptation to additional physical activity is the statistically significant interaction effect indicating that their resting HR was declining while in the controls it stayed at a steady level.

The use of the 20 mSRT results as a basis for calculating VO_2_max, as we utilized in this study, is an indirect method that is believed to be prone to error. The predictive value of the 20 mSRT results has been recently challenged by Welsman and Armstrong [30] in a study comparing the peak VO_2_ of 76 boys aged from 11–14 years obtained using a direct method and their 20 mSRT performance. The authors reported a moderate correlation between the predicted and measured VO_2_ peak, with limits of agreement close to 40% of the measured VO_2_ peak. Nevertheless, many researchers find the 20 mSRT results to be a valid, reliable, and feasible measure of the pediatric population’s CRF [31].

Following Tomkinson et al. [31], who recommend assessing CRF based on the total number of shuttles or maximum 20 mSRT speed, we analyzed both these variables in addition to participants’ VO_2_ max. The values of all three CRF indices were significantly higher in the swimmers than in the controls over the three-year study period (significant between-group effects) and, additionally, changed faster in the former. However, at no time point were the two groups significantly different from each other. The reason for this is not clear. According to Baxter-Jones and Maffulli [28], approximately 30% of an individual’s response to exercise training and physical activity depends on the genotype and the other 70% is determined by other factors. It is considered that although children are physically very active, their activity is not long and intense enough to raise their VO_2_ max [28]. In our study, the weekly duration of participants’ physical activity differed significantly between the swimmers and the controls. While the latter only had four PE lessons per week (4 × 45 min = 180 min), the swimmers also participated in swimming training sessions (180 + 4 × 70 min = 460 min per week); as a result, their total volume of physical activity was close to that recommended by WHO (420 min/week) [7]. It is possible that the reason why consecutive measurements did not find significant differences between the CRF indices of the swimmers and the controls is that swimming training is a low intensity aerobic activity.

The multiple regression analysis pointed out that the best predictors of CRF indices in the controls and the swimmers were knee breadth and the sum of four skinfold thicknesses, respectively. A follow-up regression analysis that was subsequently carried out to see which CRF indices best predicted knee breadth in the controls and the sum of four skinfold thicknesses in the swimmers indicated that these were the maximum 20 mSRT speed and VO_2_ max.

Bone breadths (knee breadth or elbow breadth) are widely used as the indexes of the so-called “frame size” [32,33]. The concept of frame size, introduced by the Metropolitan Life Insurance Company, is recommended as a reference standard for body mass. It is built on the assumption that a larger frame size involves a larger fat-free mass and a greater total body mass as a result [34]. This implies that the best predictor of the controls’ CRF could be fat-free mass. Our results are in accordance with the findings reported by Goran et al. [35], according to which fat-free mass is the strongest determinant of VO_2_ max both in children and in adults.

The fact that the sum of four skinfold thicknesses was the key predictor of the swimmers’ CRF implies the possibility of their CRF being influenced by body fatness. While this finding is a little confusing considering that Goran et al. [35] failed to find a relationship between fat mass and VO_2_ max, it is consistent with Welsman and Armstrong [30], who reported a negative correlation between the percentage body fat and maximum 20 mSRT speed. It is of note, however, that both Goran et al. [35] and Welsman and Armstrong [30] used a cross-sectional approach and did not provide any information about the level of physical activity of their subjects. Moreover, the subjects in the study by Welsman and Armstrong [30] were rather lean (the sum of the triceps and subscapular skinfolds was 18.5 ± 7.3 mm compared with 20.9 ± 10.7 mm (the swimmers) and 19.1 ± 7.1 mm (the controls) at the beginning of our study, and 20.2 ± 9.7 mm and 26.4 ± 12.5 mm at the end of it). The limited number of variables used in this study makes it difficult to explain why our findings are different from the results reported by these authors.

It is suggested that the ability of people with greater body fatness to perform aerobic-type activities (such as the 20 mSRT) is limited by insufficient submaximal aerobic capacity rather than an inadequate cardio-respiratory system [35]. This means that the amount of work an individual has to engage in during weight-bearing activities, such as the 20 mSRT, increases with fatness, causing exhaustion to come sooner. Based on the results reported by Goran et al. [35], it can be theorized that if the swimmers and the controls in our study had the same or comparable fat-free mass, the somatic predictor of CRF would be the same for both groups. However, their somatic predictors of CRF proved to be different.

There are two likely reasons for this difference: slightly greater body fat-free mass in the swimmers and/or different patterns of fat mass changes between the groups due to (1) the longitudinal character of the study; (2) the swimmers being physically more active during the three-year period than the controls; (3) the body mass and percentage of fat not differing between the groups, but a faster increase in the percentage of fat in the controls; (4) none of fatness indices in the CG being significantly correlated with CRF indices.

The study has several limitations. Firstly, although both groups differed in the volume of physical activity due to exercise training for the swimmers, the participants’ everyday activities were not subjected to a closer analysis. Therefore, we cannot exclude that these everyday activities such as active transport or non-systematic physical activities in the leisure time could affect the boys’ CRF and body fatness. Secondly, the exercise intensities during PE classes and swim training were only estimated. Since HR measurements during exercise were carried out at random, it was not possible to obtain objective data in this matter. Finally, we did not collect data on the boys’ diet and nutritional habits, which could also affect participants’ fatness. However, considering the fact that both groups of boys did not significantly differ in body mass and skinfold thicknesses it seems unlikely that caloric intake was highly diverse in these groups of boys.

## 5. Conclusions

To summarize, this longitudinal study has demonstrated that three years of swimming training as an additional physical activity did not significantly increase the selected physical traits of prepubertal boys but only slightly slowed down the rate of their biological development compared with the controls. Although the three-year values of CRF indices were significantly greater for the swimmers, the interim measurements were not different from the controls in that respect. The best predictors of the CRF of those analyzed were the knee-breadth for the controls and the sum of four skinfold thickness for the swimmers. Regarding CRF indices, the strongest correlations were found between maximum 20 mSRT speed and knee breadth in the controls and VO_2_max and the sum of four skinfold thicknesses in the swimmers. These findings suggest that three years of additional physical activity (swimming training) had a positive effect on prepubertal boys’ CRF and body fatness without significantly delaying their somatic growth.

When the shuttle run 20 m test is used to monitor the CRF of prepubertal boys over a longer period of time, their physical activity level and body fatness changes with age should be taken into account.

## Figures and Tables

**Table 1:** Arithmetic means (±SD) or medians (IQR) of the analyzed variables for the control group (con; *n* = 20) and the experimental group (swim; *n* = 20) and the results of linear regression slopes and two-way repeated measures ANOVA.

Variable		Measurement						Slope	F for Group	F for Time	F for Interaction
		1	2	3	4	5	6				
age [years]	con	10.52 ±0.31	11.00 *** ±0.31	11.48 *** ±0.32	11.96 *** ±0.31	12.41 *** ±0.31	12.94 *** ±0.30	0.480	0.1 n.s.	16,381.6 *p* < 0.001	1.6 n.s.
	swim	10.47 ±0.30	10.97 *** ±0.30	11.42 *** ±0.30	11.93 *** ±0.29	12.40 *** ±0.30	12.90 *** ±0.30	0.484			
maturity offset [years]	con	−2.49 ±0.22	−2.13 *** ±0.24	−1.80 *** ±0.27	−1.41 *** ±0.26	−1.00 *** ±0.29	−0.49 *** ±0.34	0.394	2.8 n.s.	2637.9 *p* < 0.001	1.1 n.s.
	swim	−2.62 ±0.27	−2.27 *** ±0.28	−1.97 *** ±0.29	−1.54 *** ±0.32	−1.16 *** ±0.36	−0.69 *** ±0.40	0.382			
body mass [kg]	con	37.18 ±7.20	39.12 ** ±7.33	41.00 ** ±8.28	41.35 *** (15.15)	45.79 ** ±9.68	49.19 *** ±9.84	0.024	2.8 n.s.	168.4 *p* < 0.001	1.0 n.s.
	swim	32.00 (5.80)	34.10 * (4.75)	35.05 (5.73)	36.15 *** (5.90)	38.35 ** (5.50)	44.53 *** ±9.40	0.021			
body height [m]	con	1.45 ±0.04	1.48 *** ±0.04	1.49 ** ±0.04	1.53 *** ±0.05	1.56 *** ±0.05	1.61 *** ±0.06	3.059	2.6 n.s.	413.1 *p* < 0.001	0.8 n.s.
	swim	1.424 ±0.067	1.447 *** ±0.067	1.463 * ±0.068	1.501 *** ±0.076	1.529 *** ±0.081	1.569 *** ±0.086	2.865			
BMI [kg × m^−2^]	con	17.62 ±3.00	17.90 ±3.05	18.31 ±3.31	18.67 ±3.33	17.28 (4.61)	17.98 (4.98)	0.006	1.4 n.s.	12.6 *p* < 0.001	1.3 n.s.
	swim	16.93 ±2.33	17.20 (2.73)	17.03 ±2.49	17.16 ±2.22	17.47 ±2.34	17.95 ±2.33	0.005			
body fat [%]	con	17.69 ±4.97	18.92 ±6.46	18.89 (7.88)	21.99 ±9.31	21.68 ±10.55	22.63 ±8.94	0.016	0.8 n.s.	2.2 n.s.	3.3 *p* < 0.01
	swim	16.148 (7.649)	15.758 (8.433)	15.643 (9.289)	18.427 ±6.092	15.836 (7.614)	18.154 ±6.697	−0.003			
HR_rest_ [beats × min^−1^]	con	83.20 ±9.68	81.40 ±8.34	82.40 ±9.83	82.40 ±7.50	84.00 (8.00)	83.20 ±6.17	0.001	0.9 n.s.	1.9 n.s.	3.0 *p* < 0.05
	swim	86.80 ±6.63	88.00 (6.00)	84.60 ±5.55	84.00 (4.00)	82.00 (4.00)	81.60 ±5.41	−0.006			
VO_2_max [mL × kg^−1^ × min^−1^]	con	43.08 ±2.38	43.21 ±3.21	44.62 ±4.07	44.33 ±4.24	44.59 ±4.19	45.28 (6.62)	0.002	14.6 *p* < 0.001	16.9 *p* <0.001	8.8 *p* < 0.001
	swim	46.21 ±3.75	45.98 (3.05)	48.71 ** ±6.40	48.81 ±6.05	52.59 *** (12.25)	52.29 ±5.01	0.011			
number of completed 20 mSRT shuttles	con	26.35 ±7.11	31.20 ** ±10.23	39.35 *** ±15.79	42.15 ±16.07	45.30 ±15.15	46.20 ±15.05	0.048	12.9 *p* < 0.001	95.2 *p* < 0.001	4.9 *p* < 0.001
	swim	38.85 ±14.60	42.00 (13.25)	54.40 ** ±24.67	60.20 * ±24.23	73.50 *** ±23.81	81.15 * ±20.00	0.070			
maximum 20 mSRT speed [km × h^−1^]	con	9.50 (0.13)	9.73 ±0.60	10.18 ** ±0.78	10.28 ±0.80	10.75 (1.13)	10.75 (1.13)	0.009	14.8 *p* < 0.001	73.7 *p* < 0.001	6.9 *p* < 0.001
	swim	10.15 ±0.76	10.25 (0.50)	10.98 *** ±1.26	11.15 ±1.18	12.00 *** (2.50)	12.10 ±0.97	0.016			
upper limb length [cm]	con	62.40 ±2.76	63.00 ** (3.00)	64.00 (2.00)	67.00 *** (2.25)	68.85 *** ±2.50	70.70 *** ±2.77	0.011	0.5 n.s.	190.9 *p* < 0.001	9.5 *p* < 0.001
	swim	63.30 ±3.95	63.85 ±3.86	64.00 ±3.77	65.85 *** ±4.02	66.65 * ±4.39	68.95 *** ±4.98	0.007			
lower limb length [cm]	con	87.10 ±4.09	87.45 ±4.58	89.05 * ±4.32	91.40 *** ±4.42	93.60 *** ±4.24	96.90 *** ±4.46	1.994	0.9 n.s.	167.1 *p* < 0.001	4.0 *p* < 0.01
	swim	84.80 ±4.83	87.40 *** ±4.85	88.65 ±5.05	90.90 *** ±5.64	91.50 ±5.26	94.40 *** ±5.11	1.787			
elbow breadth [mm]	con	61.75 ±8.03	68.50 *** (6.50)	73.50 *** (5.75)	76.00 (6.00)	77.00 (3.50)	78.00 (4.50)	0.020	0.1 n.s.	70.2 *p* < 0.001	23.1 *p* < 0.001
	swim	70.60 ±4.10	71.00 (5.25)	72.50 (5.00)	74.00 (6.25)	74.50 (6.75)	76.55 ±7.17	0.006			
knee breadth [mm]	con	65.50 (9.50)	71.45 *** ±5.67	75.70 *** ±6.26	78.00 ±5.92	79.40 ±6.24	79.65 ±6.40	0.017	1.6 n.s.	54.6 *p* < 0.001	20.8 *p* < 0.001
	swim	74.50 (6.75)	75.00 (5.50)	75.00 (5.50)	75.00 (5.25)	77.00 (8.50)	78.00 (8.50)	0.005			
biacromial breadth [mm]	con	336.30 ±19.99	347.80 *** ±16.09	355.75 ** ±18.47	366.15 *** ±17.45	374.70 ** ±15.60	385.00 *** ±18.22	9.560	3.4 n.s.	153.4 *p* < 0.001	5.1 *p* < 0.001
	swim	335.50 ±18.46	340.70 ±18.11	345.35 ±19.77	352.35 ±19.20	358.60 ±21.89	371.25 ±25.45	6.841			
biilio-cristal breadth [mm]	con	238.90 ±18.15	252.60 *** ±16.42	261.75 * ±22.76	270.85 * ±18.37	275.70 ±17.51	281.50 * (29.75)	0.015	0.8 n.s.	68.8 *p* < 0.001	2.5 *p* < 0.05
	swim	243.55 ±21.66	250.05 * ±18.53	256.50 ±18.27	261.50 ±18.83	268.00 ±19.78	274.50 ±19.10	0.010			
head girth [cm]	con	53.50(1.00)	54.00 **(2.00)	54.00(2.00)	54.50 (2.00)	55.00 (2.25)	55.00 (2.25)	0.002	0.1 n.s.	20.2 *p* < 0.001	0.6 n.s.
	swim	53.00 (1.50)	53.00 (2.00)	54.00 (2.00)	54.00 (3.25)	54.00 (3.00)	54.00 (3.00)	0.001			
chest girth [cm]	con	68.75 ±7.64	70.10 ±6.86	71.30 ±6.81	73.55 * ±7.35	74.75 ±7.53	76.30 ±6.98	0.009	0.4 n.s.	40.5 *p* < 0.001	0.7 n.s.
	swim	65.50 (5.75)	68.50 * (7.25)	70.55 ±7.02	70.50 (10.25)	70.50 (11.75)	74.55 ±8.29	0.008			
arm girth [cm]	con	20.00 (3.25)	21.00 * ±2.97	21.45 ±3.32	21.00 (5.25)	21.00 (6.25)	22.30 ±3.34	0.007	0.1 n.s.	34.8 *p* < 0.001	1.5 n.s.
	swim	20.00 (2.875)	20.68 ±2.47	20.90 ±2.49	21.35 ±2.70	21.30 ±2.52	22.15 ** ±2.46	0.007			
waist girth [cm]	con	64.00 ±7.82	65.60 ±7.27	67.75 ±7.66	68.95 ±8.06	70.80 ±9.28	70.80 ±8.21	0.009	0.4 n.s.	18.5 *p* < 0.001	0.7 n.s.
	swim	63.80 ±7.04	64.65 ±7.83	65.65 ±7.96	65.50 (4.50)	66.00 (12.75)	69.75 ±6.75	0.008			
thigh girth [cm]	con	36.95 ±4.77	37.80 ±3.83	38.10 ±3.77	40.35 ** ±3.72	41.35 ±4.40	42.10 ±4.60	1.104	1.5 n.s.	21.8 *p* < 0.001	6.1 *p* < 0.001
	swim	36.13 ±4.28	37.40 ±4.06	38.65 ±4.68	38.00 ±4.44	38.90 ±4.86	38.45 ±4.95	0.442			
subsca-pular skinfold [mm]	con	5.75 (3.23)	5.80 (3.48)	7.05 (8.10)	7.40 (5.95)	6.65 (10.80)	6.45 (10.23)	0.026	0.1 n.s.	2.3 *p* < 0.05	2.3 *p* < 0.05
	swim	6.90 (3.20)	5.65 (5.53)	5.95 (5.03)	6.45 (6.40)	5.85 (4.73)	6.35 (3.08)	−0.003			
suprailiac skinfold [mm]	con	7.45 (7.83)	8.75 (15.05)	11.05 (13.88)	13.10 (19.50)	14.57 ±9.67	13.90 (11.15)	0.031	1.2 n.s.	7.5 *p* < 0.001	1.9 n.s.
	swim	6.75 (5.73)	7.80 (8.25)	9.10 (8.58)	8.75 (8.18)	8.85 (10.25)	9.85 ±4.19	0.009			
biceps skinfold [mm]	con	7.20 ±3.68	7.24 ±3.75	7.38 ±4.37	7.92 ±4.15	5.50 (5.28)	7.77 ±3.18	0.010	0.6 n.s.	0.8 n.s.	3.6 *p* < 0.01
	swim	6.45 (4.75)	7.01 ±3.21	7.40 ±3.72	6.33 ±2.88	5.88 ±2.81	5.75 (3.03)	−0.026			
triceps skinfold [mm]	con	12.21 ±4.83	13.24 ±5.20	14.76 ±6.78	15.77 ±7.15	15.78 ±8.62	16.10 ±7.12	0.020	1.4 n.s.	1.1 n.s.	1.9 n.s.
	swim	11.00 (7.70)	12.24 ±5.84	13.20 ±7.12	12.13 ±5.16	11.86 ±4.57	12.18 ±5.58	−0.003			
sum of 4 skinfolds [mm]	con	36.81 ±16.27	40.30 ±19.51	40.10 (27.90)	44.00 (43.95)	47.39 ±25.70	49.34 ±22.31	0.023	1.0 n.s.	4.2 *p* < 0.01	5.0 *p* < 0.001
	swim	28.80 (19.85)	28.20 (25.75)	32.25 (31.28)	30.00 (29.63)	28.90 (25.43)	36.21 ±16.77	−0.004			

*—statistically different from the previous measurement (*—*p* < 0.05; **—*p* < 0.01; ***—*p* < 0.001). Abbreviations: BMI—body mass index; HR_rest_—resting heart rate; VO_2_max—maximum oxygen uptake; sum of 4 skinfolds—sum of subscapular, suprailiac, biceps and triceps skinfold thicknesses.

**Table 2:** Pearson’s correlation coefficients between the number of completed 20 mSRT shuttles and biological variables for the control group (con; *n* = 120) and the experimental group (swim; *n* = 120) adjusted for the false discovery rate of 0.1.

Variable	CON No. of Completed 20 mSRT Shuttles		Variable	SWIM No. of Completed 20 mSRT Shuttles		Benjamini-Hochberg Critical Value
	R	*p*		R	*p*	
knee breadth [mm]	0.478	3.390 × 10^−8^ significant	age [years]	0.527	6.188 × 10^−10^ significant	0.005
age [years]	0.345	1.155 × 10^−4^ significant	biceps skinfold [mm]	−0.462	1.085 × 10^−7^ significant	0.009
elbow breadth [mm]	0.340	1.470 × 10^−4^ significant	sum of 4 skinfolds [mm]	−0.420	1.849 × 10^−6^ significant	0.014
biiliocristal breadth [mm]	0.259	4.257 × 10^−3^ significant	body fat [%]	−0.390	1.056 × 10^−5^ significant	0.018
thigh girth [cm]	0.217	1.710 × 10^−2^ significant	triceps skinfold [mm]	−0.390	1.092 × 10^−5^ significant	0.023
biacromial breadth [mm]	0.216	1.811 × 10^−2^ significant	biacromial breadth [mm]	0.380	1.843 × 10^−5^ significant	0.027
upper limb length [cm]	0.193	3.446 × 10^−2^ n.s.	upper limb length [cm]	0.361	5.093 × 10^−5^ significant	0.032
subscapular skinfold [mm]	0.177	5.359 × 10^−2^ n.s.	subscapular skinfold [mm]	−0.346	1.085 × 10^−4^ significant	0.036
body fat [%]	0.175	5.530 × 10^−2^ n.s.	body height [m]	0.345	1.124 × 10^−4^ significant	0.041
triceps skinfold [mm]	0.171	6.176 × 10^−2^ n.s.	suprailliac skinfold [mm]	−0.342	1.309 × 10^−4^ significant	0.045
sum of 4 skinfolds [mm]	0.163	7.459 × 10^−2^ n.s.	HR_rest_ [beats × min^−1^]	−0.335	1.837 × 10^−4^ significant	0.050
body height [m]	0.159	8.277 × 10^−2^ n.s.	elbow breadth [mm]	0.326	2.850 × 10^−4^ significant	0.055
body mass [kg]	0.158	8.433 × 10^−2^ n.s.	lower limb length [cm]	0.320	3.688 × 10^−4^ significant	0.059
suprailliac skinfold [mm]	0.154	9.309 × 10^−2^ n.s.	biiliocristal breadth [mm]	0.169	6.454 × 10^−2^ n.s.	0.064
lower limb length [cm]	0.152	9.669 × 10^−2^ n.s.	body mass [kg]	0.156	8.895 × 10^−2^ n.s.	0.068
HR_rest_ [beats × min^−1^]	−0.139	1.308 × 10^−1^ n.s.	thigh girth [cm]	−0.143	1.204 × 10^−1^ n.s.	0.073
biceps skinfold [mm]	0.124	1.780 × 10^−1^ n.s.	head girth [cm]	0.136	1.402 × 10^−1^ n.s.	0.077
BMI [kg × m^2^]	0.113	2.201 × 10^−1^ n.s.	knee breadth [mm]	0.108	2.408 × 10^−1^ n.s.	0.082
chest girth [cm]	0.069	4.575 × 10^−1^ n.s.	BMI [kg × m^2^]	−0.070	4.467 × 10^−1^ n.s.	0.086
arm girth [cm]	0.067	4.687 × 10^−1^ n.s.	chest girth [cm]	0.051	5.819 × 10^−1^ n.s.	0.091
waist girth [cm]	−0.019	8.342 × 10^−1^ n.s.	arm girth [cm]	−0.047	6.105 × 10^−1^ n.s.	0.095
head girth [cm]	0.008	9.311 × 10^−1^ n.s.	waist girth [cm]	0.044	6.338 × 10^−1^ n.s.	0.100

**Table 3:** Pearson’s correlation coefficients between the maximum 20 mSRT speed and biological variables for the control group (con; *n* = 120) and the experimental group (swim; *n* = 120) adjusted for the false discovery rate of 0.1.

Variable	CON Maximum 20 mSRT Speed		Variable	SWIM Maximum 20 mSRT Speed		Benjamini-Hochberg Critical Value
	R	*p*		R	*p*	
knee breadth [mm]	0.480	2.910 × 10^−8^ significant	age [years]	0.507	3.328 × 10^−9^ significant	0.005
age [years]	0.347	1.037 × 10^−4^ significant	biceps skinfold [mm]	−0.466	7.910 × 10^−8^ significant	0.009
elbow breadth [mm]	0.344	1.188 × 10^−4^ significant	sum of 4 skinfolds [mm]	−0.407	3.951 × 10^−6^ significant	0.014
biiliocristal breadth [mm]	0.255	4.892 × 10^−3^ significant	biacromial breadth [mm]	0.387	1.279 × 10^−5^ significant	0.018
thigh girth [cm]	0.214	1.909 × 10^−2^ significant	upper limb length [cm]	0.374	2.641 × 10^−5^ significant	0.023
biacromial breadth [mm]	0.210	2.136 × 10^−2^ significant	triceps skinfold [mm]	−0.368	3.531 × 10^−5^ significant	0.027
triceps skinfold [mm]	0.190	3.721 × 10^−2^ n.s.	body fat [%]	−0.368	3.547 × 10^−5^ significant	0.032
upper limb length [cm]	0.186	4.167 × 10^−2^ n.s.	elbow breadth [mm]	0.357	6.156 × 10^−5^ significant	0.036
body fat [%]	0.184	4.474 × 10^−2^ n.s.	body height [m]	0.355	6.914 × 10^−5^ significant	0.041
sum of 4 skinfolds [mm]	0.170	6.283 × 10^−2^ n.s.	HR_rest_ [beats × min^−1^]	−0.353	7.642 × 10^−5^ significant	0.045
suprailliac skinfold [mm]	0.162	7.766 × 10^−2^ n.s.	subscapular skinfold [mm]	−0.332	2.137 × 10^−4^ significant	0.050
subscapular skinfold [mm]	0.161	7.854 × 10^−2^ n.s.	suprailliac skinfold [mm]	−0.331	2.193 × 10^−4^ significant	0.055
body height [m]	0.146	1.129 × 10^−1^ n.s.	lower limb length [cm]	0.327	2.706 × 10^−4^ significant	0.059
body mass [kg]	0.143	1.180 × 10^−1^ n.s.	biiliocristal breadth [mm]	0.192	3.599 × 10^−2^ significant	0.064
lower limb length [cm]	0.138	1.319 × 10^−1^ n.s.	body mass [kg]	0.177	5.332 × 10^−2^ significant	0.068
HR_rest_ [beats × min^−1^]	−0.131	1.523 × 10^−1^ n.s.	head girth [cm]	0.148	1.058 × 10^−1^ n.s.	0.073
biceps skinfold [mm]	0.123	1.805 × 10^−1^ n.s.	thigh girth [cm]	−0.123	1.806 × 10^−1^ n.s.	0.077
BMI [kg × m^2^]	0.101	2.707 × 10^−1^ n.s.	knee breadth [mm]	0.103	2.646 × 10^−1^ n.s.	0.082
arm girth [cm]	0.067	4.646 × 10^−1^ n.s.	chest girth [cm]	0.067	4.651 × 10^−1^ n.s.	0.086
chest girth [cm]	0.064	4.864 × 10^−1^ n.s.	BMI [kg × m^2^]	−0.047	6.119 × 10^−1^ n.s.	0.091
waist girth [cm]	−0.015	8.714 × 10^−1^ n.s.	waist girth [cm]	0.045	6.261 × 10^−1^ n.s.	0.095
head girth [cm]	0.013	8.902 × 10^−1^ n.s.	arm girth [cm]	−0.020	8.296 × 10^−1^ n.s.	0.100

**Table 4:** Pearson’s correlation coefficients between the maximum oxygen uptake values (VO_2_max) predicted on the basis of 20 mSRT and investigated biological variables in the control group (con; *n* = 120) and the experimental group (swim; *n* = 120) with adjustments for the false discovery rate of 0.1.

Variable	CON Maximum 20 mSRT Speed		Variable	SWIM Maximum 20 mSRT Speed		Benjamini-Hochberg Critical Value
	r	*p*		r	*p*	
knee breadth [mm]	0.259	4.241 × 10^−3^ significant	biceps skinfold [mm]	−0.467	7.810 × 10^−8^ significant	0.005
waist girth [cm]	−0.169	6.534 × 10^−2^ n.s.	sum of 4 skinfolds [mm]	−0.430	9.600 × 10^−7^ significant	0.009
HR_rest_ [beats × min^−1^]	−0.147	1.103 × 10^−1^ n.s.	triceps skinfold [mm]	−0.388	1.172 × 10^−5^ significant	0.014
body height [m]	−0.139	1.309 × 10^−1^ n.s.	body fat [%]	−0.381	1.740 × 10^−5^ significant	0.018
elbow breadth [mm]	0.122	1.848 × 10^−1^ n.s.	suprailliac skinfold [mm]	−0.371	2.997 × 10^−5^ significant	0.023
triceps skinfold [mm]	0.106	2.496 × 10^−1^ n.s.	subscapular skinfold [mm]	−0.347	1.026 × 10^−4^ significant	0.027
upper limb length [cm]	−0.103	2.616 × 10^−1^ n.s.	HR_rest_ [beats × min^−1^]	−0.336	1.735 × 10^−4^ significant	0.032
body fat [%]	0.096	2.976 × 10^−1^ n.s.	upper limb length [cm]	0.335	1.856 × 10^−4^ significant	0.036
chest girth [cm]	−0.091	3.229 × 10^−1^ n.s.	biacromial breadth [mm]	0.328	2.516 × 10^−4^ significant	0.041
lower limb length [cm]	−0.084	3.635 × 10^−1^ n.s.	elbow breadth [mm]	0.324	3.036 × 10^−4^ significant	0.045
subscapular skinfold [mm]	0.078	3.976 × 10^−1^ n.s.	age [years]	0.314	4.862 × 10^−4^ significant	0.050
sum of 4 skinfolds [mm]	0.075	4.172 × 10^−1^ n.s.	body height [m]	0.279	1.997 × 10^−3^ significant	0.055
biacromial breadth [mm]	−0.069	4.525 × 10^−1^ n.s.	lower limb length [cm]	0.260	4.193 × 10^−3^ significant	0.059
suprailliac skinfold [mm]	0.066	4.747 × 10^−1^ n.s.	head girth [cm]	0.184	4.443 × 10^−2^ significant	0.064
biceps skinfold [mm]	0.058	5.325 × 10^−1^ n.s.	thigh girth [cm]	−0.174	5.773 × 10^−2^ significant	0.068
age [years]	−0.043	6.399 × 10^−1^ n.s.	body mass [kg]	0.118	2.014 × 10^−1^ n.s.	0.073
arm girth [cm]	−0.043	6.451 × 10^−1^ n.s.	biiliocristal breadth [mm]	0.112	2.215 × 10^−1^ n.s.	0.077
body mass [kg]	−0.039	6.749 × 10^−1^ n.s.	knee breadth [mm]	0.082	3.732 × 10^−1^ n.s.	0.082
thigh girth [cm]	0.038	6.794 × 10^−1^ n.s.	BMI [kg × m^2^]	−0.070	4.462 × 10^−1^ n.s.	0.086
BMI [kg × m^2^]	0.028	7.650 × 10^−1^ n.s.	arm girth [cm]	−0.065	4.834 × 10^−1^ n.s.	0.091
biiliocristal breadth [mm]	0.016	8.666 × 10^−1^ n.s.	chest girth [cm]	0.015	8.711 × 10^−1^ n.s.	0.095
head girth [cm]	−0.014	8.831 × 10^−1^ n.s.	waist girth [cm]	−0.013	8.864 × 10^−1^ n.s.	0.100

**Table 5:** The results of a stepwise multiple regression analysis with backward elimination for dependent variables: number of completed 20 mSRT shuttles, maximum 20 mSRT speed, and maximum oxygen uptake (VO_2_max) predicted on the basis of 20 mSRT.

Dependent Variable		R^2^	SEE	Independent Variable	ß ±SE of ß	B ±SE of B	*p*
number of completed 20 mSRT shuttles	con	0.228 *p* < 0.001	±0.157	intercept	-	−1.668 ±0.545	<0.01
				knee breadth	0.478 ±0.081	1.719 ±0.291	<0.001
	swim	0.176 *p* < 0.001	±0.188	intercept	-	2.349 ±0.126	<0.001
				sum of 4 skinfolds	−0.420 ±0.084	−0.410 ±0.082	<0.001
maximum 20 mSRT speed [km × h^−1^]	con	0.230 *p* < 0.001	±0.029	intercept	-	0.397 ±0.102	<0.001
				knee breadth	0.480 ±0.081	0.324 ±0.055	<0.001
	swim	0.166 *p* < 0.001	±0.044	intercept	-	1.186 ±0.030	<0.001
				sum of 4 skinfolds	−0.407 ±0.084	−0.094 ±0.019	<0.001
VO_2_max [mL × kg^−1^ × min^−1^]	con	0.067 *p* < 0.01	±0.036	intercept	-	1.278 ±0.125	<0.001
				knee breadth	0.259 ±0.089	0.194 ±0.067	<0.01
	swim	0.185 *p* < 0.001	±0.046	intercept	-	1.845 ±0.031	<0.001
				sum of 4 skinfolds	−0.430 ±0.083	−0.103 ±0.020	<0.001

**Table 6:** The results of a stepwise multiple regression analysis with backward elimination for dependent variables: knee breadth and the sum of four skinfold thicknesses (subscapular, suprailiac, biceps, and triceps) in regard to the number of running shuttles performed during the 20 mSRT, maximum 20 mSRT speed, and VO_2_max calculated of the basis of results of 20 mSRT as independent variables for control and swimming group, respectively.

Dependent Variable		R^2^	SEE	Independent Variable	ß ±SE of ß	B ±SE of B	*p*
knee breadth [mm]	con	0.230 *p* < 0.001	0.044	intercept	-	1.158 ±0.120	<0.001
				20 mSRT maximum speed	0.480 ±0.081	0.711 ±0.120	<0.001
sum of 4 skinfolds [mm]	swim	0.185 *p* < 0.001	0.191	intercept	-	4.562 ±0.587	<0.001
				VO_2_max	−0.430 ±0.083	−1.798 ±0.348	<0.001

## Data Availability

The data presented in this study are available upon request from the corresponding author.
